# Supplementary figures and images for: Transcranial Alternating Current Stimulation (tACS) for patients with Post-Stroke Anomia: Preliminary Data on Picture Naming Performance
Source: PLoS One. 2026 Feb 23;21(2):e0342191. doi: 10.1371/journal.pone.0342191 (PMC12928461; doi:10.1371/journal.pone.0342191)

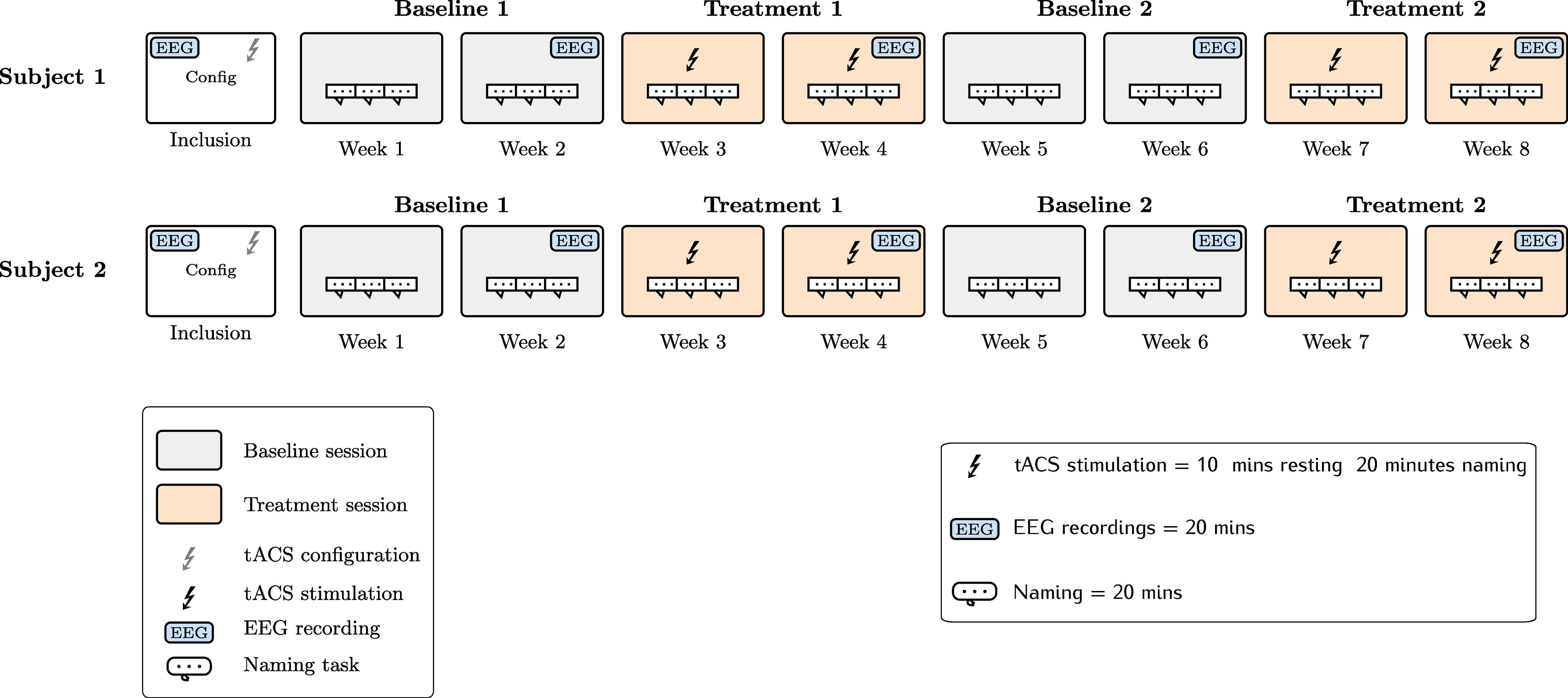

Supplement: S1 Fig — Each session included a fixed sequence of task blocks, with stimulation restricted to shaded intervention blocks and EEG acquired at predefined points to compare baseline, intervention, and washout activity. (TIF) [file pone.0342191.s001.tif]

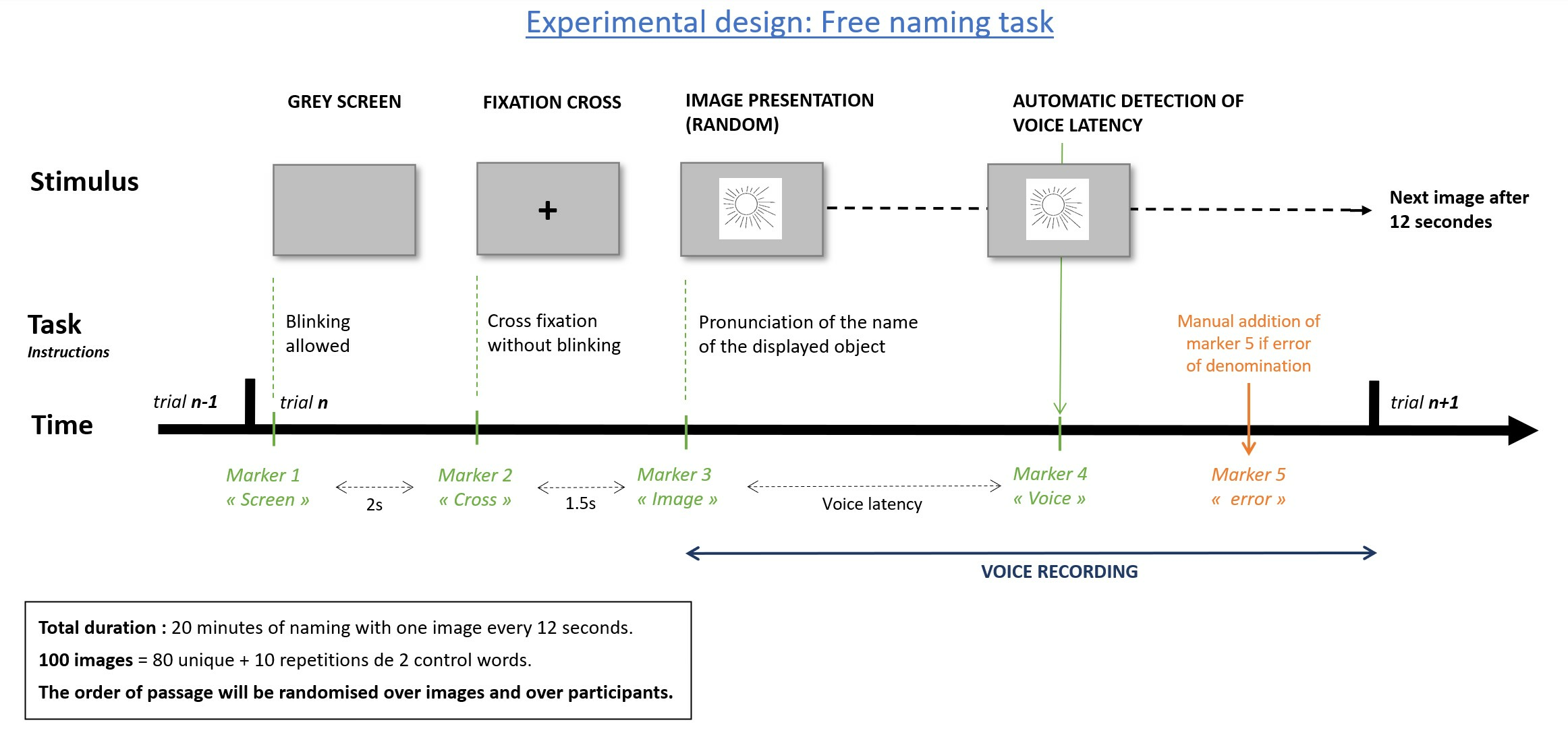

Supplement: S2 Fig — Sequence within each trial: grey screen with free blinking, fixation cross without blinking, random image presentation, overt naming, automatic detection of voice onset, and optional marking of naming errors. Markers 1–5 on the timeline indicate the onset of the grey screen, fixation cross, image, voice response, and experimenter-annotated errors, respectively; one image is presented every 12 s (100 images over ~20 min, in randomized order) while vocal responses are continuously. (TIF) [file pone.0342191.s002.tif]

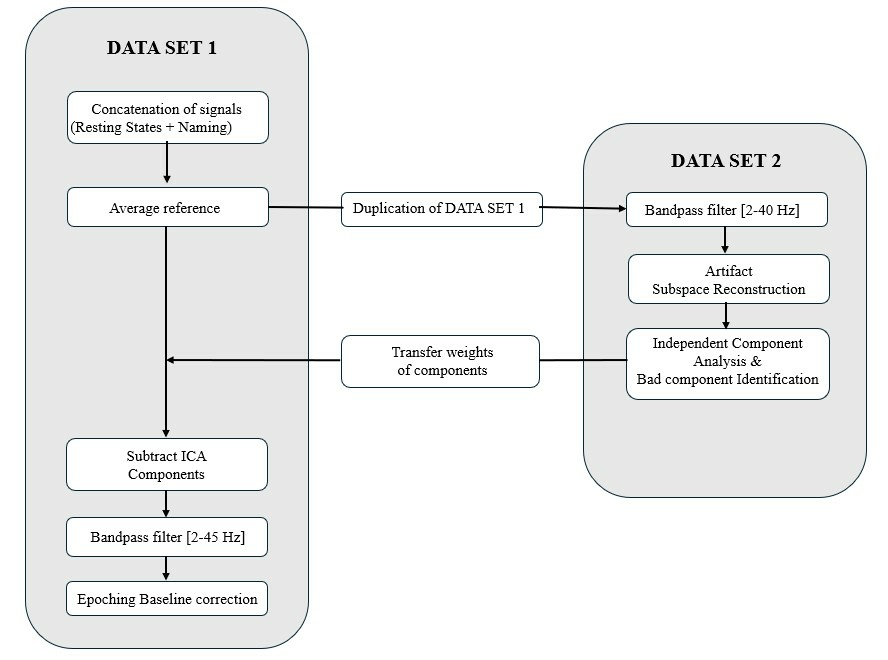

Supplement: S3 Fig — Data set 1 is average-referenced and duplicated into Data set 2 for band-pass filtering (2–40 Hz), Artifact Subspace Reconstruction, ICA and bad-component identification; the resulting component weights are then transferred back to Data set 1 for artifact subtraction, followed by band-pass filtering (2–45 Hz), epoching, and baseline correction. (TIF) [file pone.0342191.s003.tif]

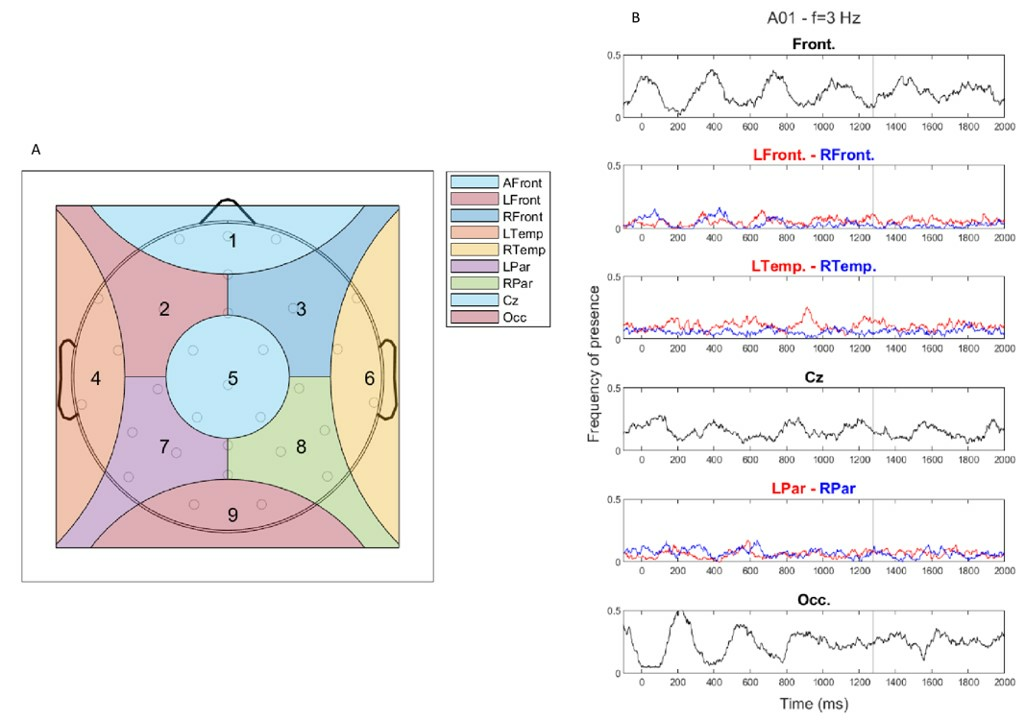

Supplement: S4 Fig — A) Scalp parcellation. B) Distribution of the maxima of amplitude across 100 trials at a frequency of 3 Hz. 0 indicates the appearance of the image. The vertical line indicates the median latency over all trials. The Y axis indicates the frequency of presence across the 100 trials and the x axis indicates time values in milliseconds. (TIF) [file pone.0342191.s004.tif]

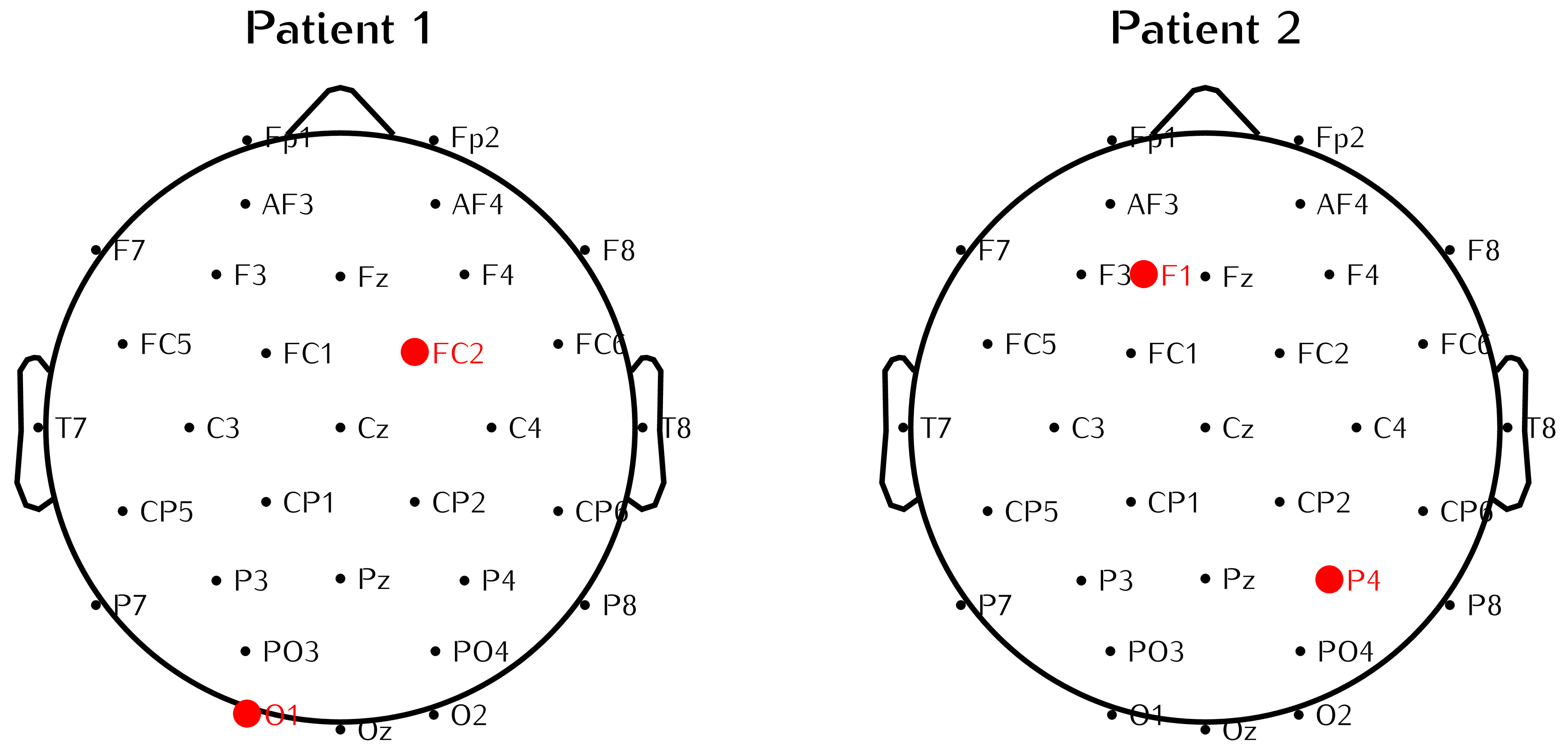

Supplement: S5 Fig — Red circles indicate the sites selected for stimulation (Patient 1: FC2 and O1; Patient 2: F1 and P4). (TIF) [file pone.0342191.s005.tif]

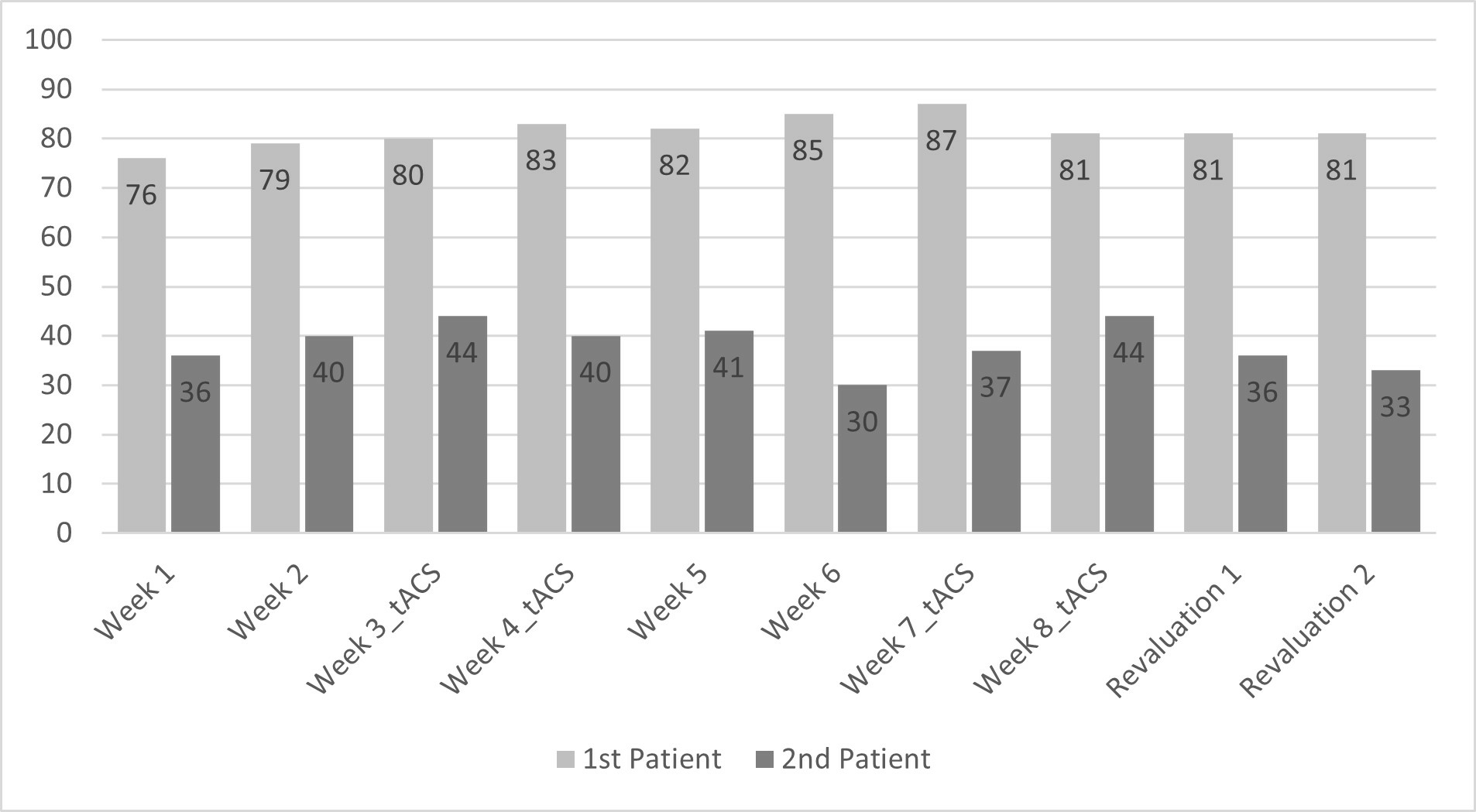

Supplement: S6 Fig — Weeks 3, 4, 7, and 8 with tACS stimulation and the first and second follow-up re-evaluations. (TIF) [file pone.0342191.s006.tif]

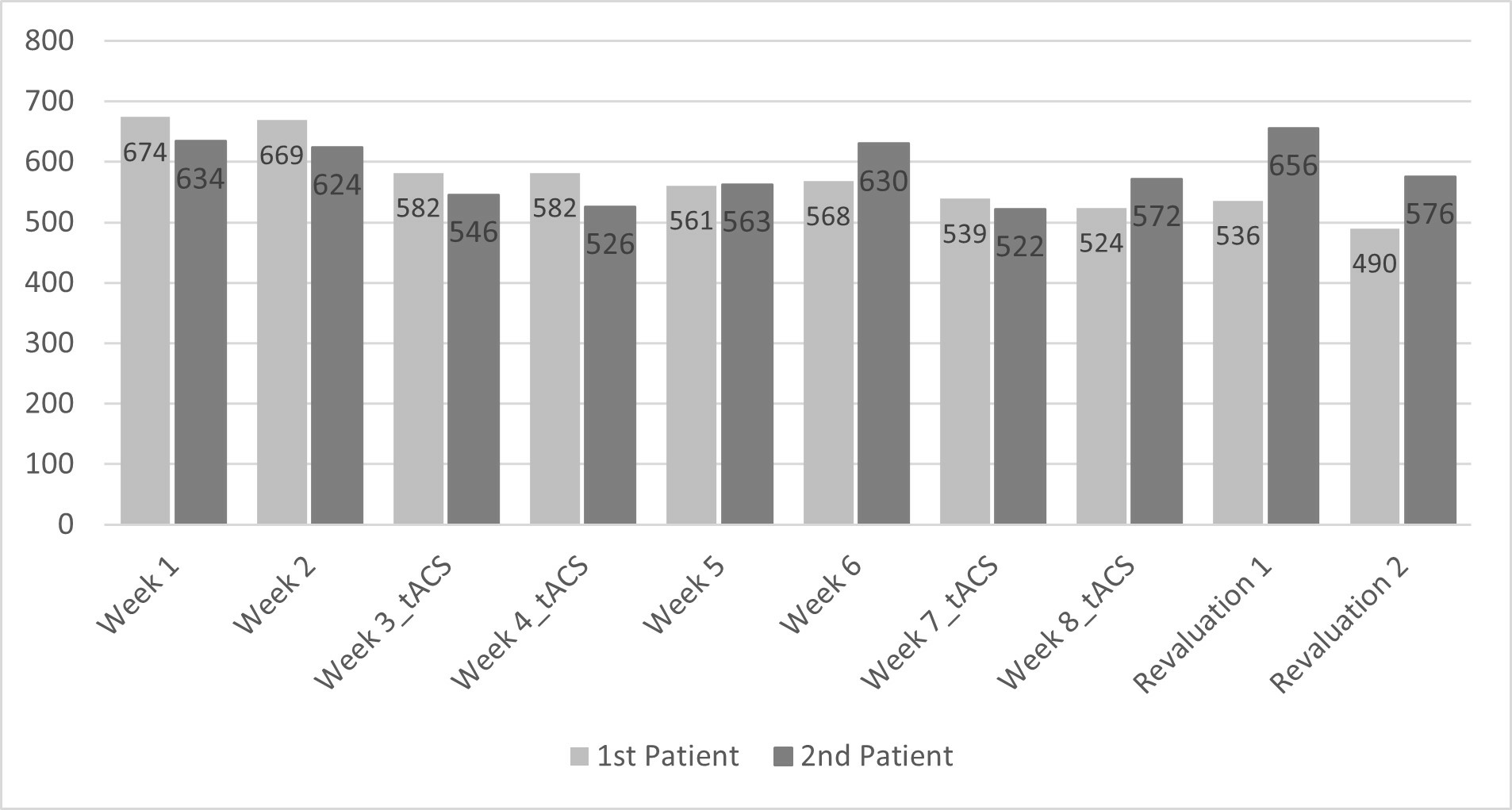

Supplement: S7 Fig — Weekly mean naming latencies (ms) on the picture-naming task for each patient (Weeks 1–8), including weeks with tACS stimulation (Weeks 3, 4, 7, and 8). Bars for the first and second follow-up re-evaluations show naming latencies after the intervention period. (TIF) [file pone.0342191.s007.tif]

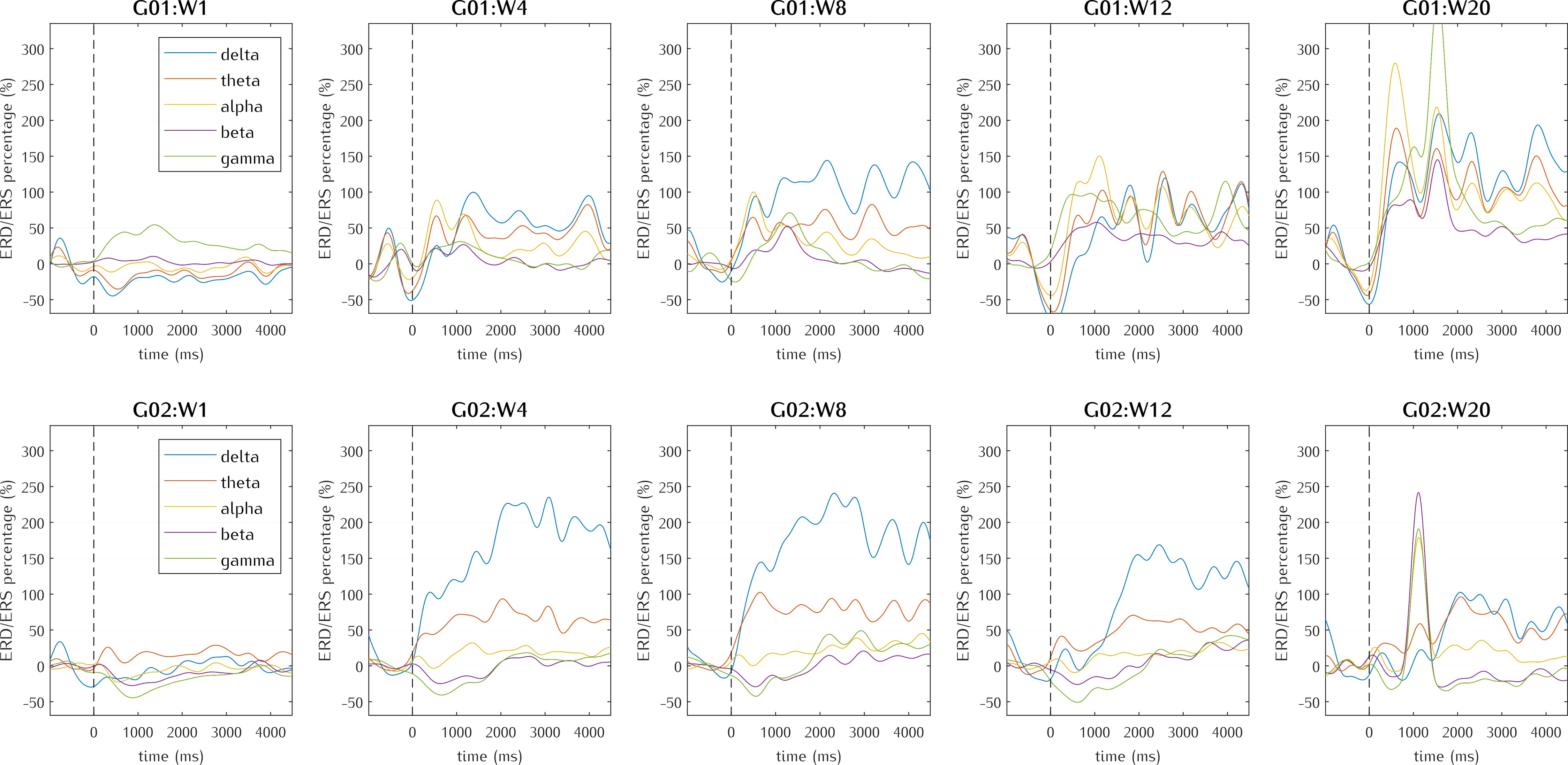

Supplement: S8 Fig — The end of the first (W1), the median (W4), the last week of the intervention (W8) and follow-up assessments one (W12) and three months (W20) after completion of the intervention. (TIF) [file pone.0342191.s008.tif]

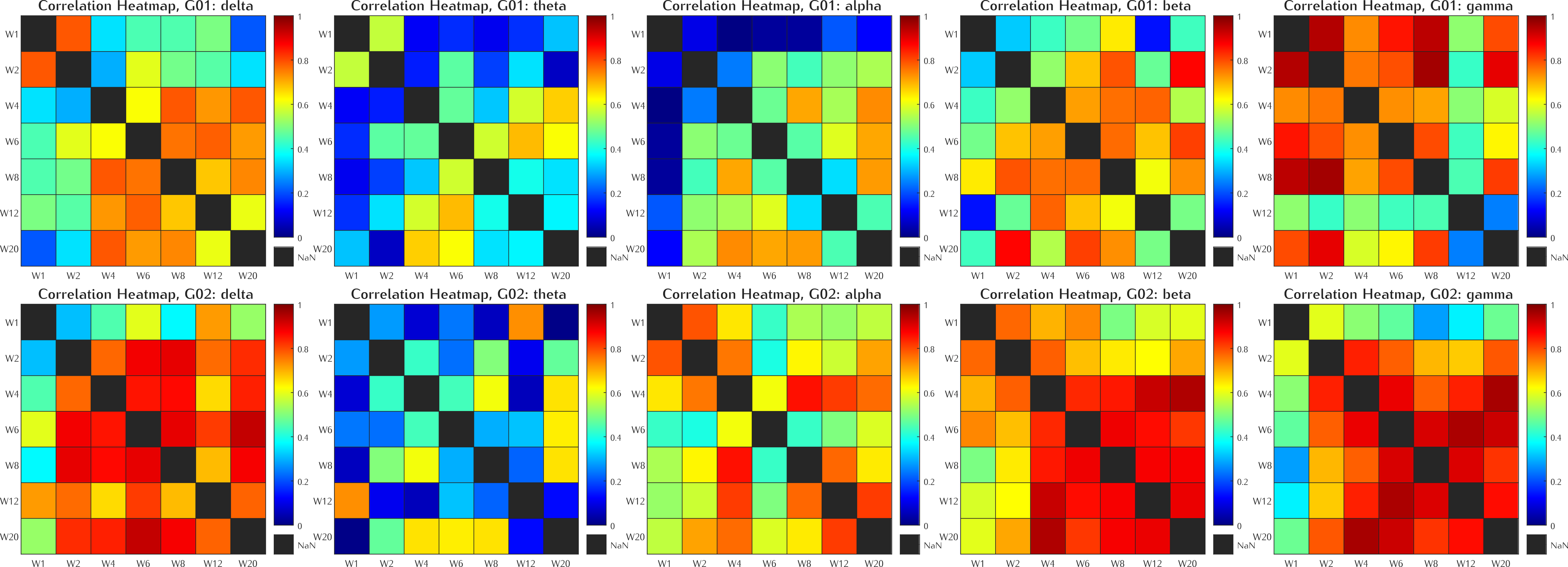

Supplement: S9 Fig — Pearson correlation matrices of band-limited power spectra (delta, theta, alpha, beta, gamma) across seven EEG sessions (W1, W2, W4, W6, W8, W12, W20) for Patient 1 (G01, top row) and Patient 2 (G02, bottom row). Each heatmap displays pairwise correlations (0–1) between sessions for a given frequency band, with diagonal elements (self-correlations) set to NaN. (TIF) [file pone.0342191.s009.tif]

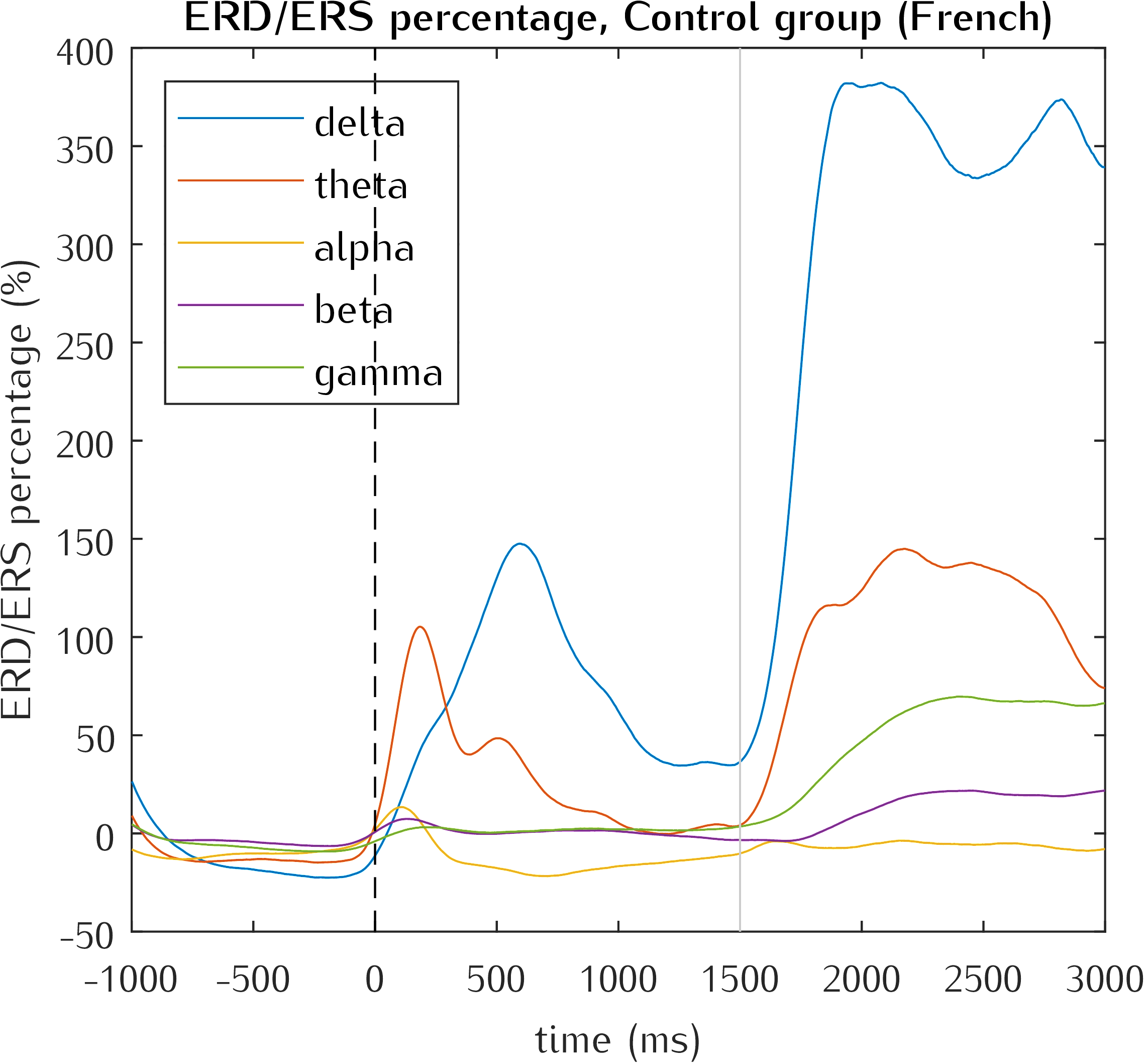

Supplement: S10 Fig — Event-related desynchronization/synchronization (ERD/ERS) time courses for delta, theta, alpha, beta, and gamma bands in 16 healthy control subjects during the forced picture-naming task. ERD/ERS percentage is plotted relative to baseline and time-locked to picture onset (0 ms, dashed line) and to the go-signal for naming at 1500 ms (grey line). (TIF) [file pone.0342191.s010.tif]
